# Inflammation to cancer: The molecular biology in the pancreas (Review)

**DOI:** 10.3892/ol.2014.2003

**Published:** 2014-03-28

**Authors:** SUNBIN LING, TINGTING FENG, KAIQI JIA, YU TIAN, YAN LI

**Affiliations:** 1 Department of General Surgery, The Second Affiliated Hospital of Dalian Medical University, Dalian, Liaoning 116023, P.R. China; 2 Department of Medical Oncology, The Second Affiliated Hospital of Dalian Medical University, Dalian, Liaoning 116023, P.R. China; 3 Institute of Cancer Stem Cells, Dalian Medical University, Dalian, Liaoning 116044, P.R. China; 4 College of Basic Medical Sciences, Dalian Medical University, Dalian, Liaoning 116044, P.R. China

**Keywords:** inflammation, pancreatic cancer, K-ras, nuclear factor-κB, cyclooxygenase-2, serine protease inhibitor kazal type 1, reactive oxygen species, proinflammatory cytokines

## Abstract

Inflammatory responses are known to be correlated with cancer initiation and progression, and exploration of the route from inflammation to cancer makes a great contribution in elucidating the mechanisms underlying cancer development. Pancreatic cancer (PC) is a lethal disease with a low radical-resection rate and a poor prognosis. As chronic pancreatitis is considered to be a significant etiological factor for PC development, the current review aims to describe the molecular pathways from inflammation to pancreatic carcinogenesis, in support of the strategies for the prevention, diagnosis and treatment of PC.

## 1. Introduction

Pancreatic cancer (PC), for which the overall 5-year survival rate among patients is ~6% (1), has the fourth highest incidence ratio of cancer-related fatalities, with <10% of patients having the opportunity of radical surgery at diagnostic presentation. Current evidence has indicated that the associated risk factors include smoking, alcoholism, diabetes mellitus, genetic factors and chronic pancreatitis (CP) (2–6). However, the causes and mechanisms of PC remain unclear.

Inflammatory responses play a significant role in cancer development, including the initial malignant conversion. The potential association between inflammation and cancer has been observed in various types of malignancies, including persistent *Helicobacter pylori* infection with gastric carcinoma (7), inflammatory bowel disease with colorectal cancer (8,9) and reflux esophagitis with esophageal adenocarcinoma (10). It is widely believed that tumor initiation is triggered by multiple mutational hits that induce DNA damage and genomic instability. The potential carcinogens produced from inflammatory cells, such as reactive oxygen species (ROS) and reactive nitrogen intermediates, are capable of inducing malignant initiation through accumulation of continuous DNA damage and subsequent abortive repair. Furthermore, the production of growth factors and cytokines during the inflammatory process can enhance the proliferation of initiated cells by eventually converting them to tumor cells (11). Assessing the link between chronic inflammation and tumorigenesis may provide a different approach to understanding the pathological mechanisms of tumor development. Accordingly, the present review discusses the molecular biology of CP to PC, which may contribute to furthering the clinical diagnosis and therapy.

## 2. Chronic pancreatitis and risk of pancreatic cancer

Several studies have demonstrated a strong link between antecedent CP and PC. The incidence is variable and the standardized incidence ratio (SIR) has been reported to be between 3.8 (12) and 18.5 (13). In 1993, Lowenfels *et al* (14) launched an international cohort study with 2,015 patients, demonstrating that the SIR was 14.4. The risk of malignant transformation 10 and 20 years after diagnosis was 1.8 and 4.0%, respectively. However, certain epidemiological factors, including cigarette smoking and alcohol (15), which are considered as cofactors in the development of pancreatitis, are also responsible for the increased PC incidence (6,16).

Additionally, two recent large-sample case-control studies of PC identified that a short temporal history of pancreatitis was highly associated with PC (17), indicating that pancreatitis may be an early manifestation of PC in specific individuals. Patients with PC may be diagnosed with CP in the first diagnosis, while the differential diagnosis between early CP and PC is difficult. The misdiagnosis may elevate the correlation of the two.

However, with adjustments or matching variables, including smoking habit and alcohol consumption, the risk of PC increases in patients with CP (18). In patients with hereditary pancreatitis, the lifetime risk of PC is ~40% (19), and similarly, for those patients with tropical pancreatitis, the risk of PC is also high (20). Furthermore, a study by Talamini *et al* (13) analyzed 715 cases of CP with a median follow-up of 10 years, observing that the risks of PC and non-PC were increased as compared with the general population. Notably, the clearly higher incidence of PC indicated that although cigarette smoking contributed to various tumors, there were certain other factors linked to the chronic inflammation of the pancreas, which may be responsible for the increased risk.

In summary, CP is determinately considered as an independent risk factor for PC. This risk is markedly increased in those patients with hereditary pancreatitis or tropical pancreatitis.

## 3. Molecular pathway between chronic pancreatitis and cancer of the pancreas

With the documented link between CP and PC, the molecular pathway from inflammation to cancer in the pancreas exhibits an increasing significance for deeper analysis. Novel molecular changes have been observed in CP and PC simultaneously, including K-ras mutations (21,22) and serine protease inhibitor Kazal type 1 (SPINK1) gene N34S mutations (23,24). In addition, the cyclooxygenase-2 (COX-2) enzyme and nitric oxide (NO), which are considered to be inflammatory mediators and have been found to be overexpressed in patients with CP (25,26), may act as key factors in the tumorigenesis in CP. Therefore, further discussion in the present review focuses on the roles of novel molecules in pancreatic tumorigenesis.

### 3.1. K-ras

The Ras family, which consists of three members, known as H-, N- and K-ras, are proto-oncogenes that encode a highly-homologous group of 21-kDa monomeric, membrane-localized guanosine triphosphate (GTP)-ases. The main function of these proteins is to transmit signals between the extracellular and intracellular environment, acting as a ‘molecular switch’. As downstream molecules with normal regulation sustaining the homeostasis associated with cell proliferation and apoptosis, the error state-like point mutation, multiple expression, insertion and transposition of the Ras genes are responsible for the carcinogenesis of numerous organs.

The K-ras gene is located on chromosome 12p12, and is ~45,000 bp in length. K-ras is the most cancer-related protein in the p21 Ras proteins, existing in two alternatively spliced forms, K-Ras4A and K-Ras4B, which have differing c-terminal residues resulting in differential post-translational modification. Mutations frequently detected in codon 12, 13 and 61 of the K-ras gene result in the subsequent translational product remaining in the GTP-bound (27), activated state, which may activate the downstream pathways, including Raf/MEK/extracellular signal-regulated kinase (ERK) (28–30) and Ras/phosphatidylinositol-3-kinase (PI3K)/Akt (31,32). The complex intracellular regulations originating from the K-ras mutation finally result in a proliferation and apoptosis-related malignant transformation in certain cells (32,33). Constitutive activation of K-ras has been observed in specific pathological changes in the pancreas, including CP, pancreatic intraepithelial neoplasia (PanIN) and PC (34), among which the simultaneous overexpression of K-ras exhibiting the potential linkage, has attracted widespread attention. Mu *et al* (35) investigated the clinical significance of K-ras gene mutation detection in patients with CP and observed that screening for the K-ras mutation may be useful in identifying patients with pancreatitis that are at a high risk for developing cancer. In 2007, Guerra *et al* (36) established a mouse model with CP induced by caerulein treatment, and observed that the chronic inflammation in the pancreas could facilitate the differentiation of acinar/centroacinar or their precursor cells into ductal-like cells, resulting in PanINs and pancreatic ductal adenocarcinoma (PDA) by the selective expression of an endogenous K-RasG12V oncogene in adult mice. The formation of PanINs followed a defined progression from low- to high-grade lesions that resulted in the appearance of invasive PDA by completely depending on K-RasG12Vgeo expression, as reported similarly in other studies (21,37). Guerra *et al* (36) also assessed the cooperation between K-RasG12V expression and p53 inactivation, observing that the additional mutation of p53 could strongly enhance the metastatic properties of K-RasG12V-induced PDA. As reviewed in these studies, it is now accepted that the K-Ras oncogene forms the linkage between CP and PDA. Recently, Guerra *et al* (38) completed a further study to approach the molecular pathways from CP to PC with expression of a resident K-ras oncogene in adult mice. The study demonstrated that the K-ras mutation could initiate murine PanINs (mPanINs) and murine PDAC (mPDAC) in adult mice with pre-existing pancreatic damage and an inflammatory response. Furthermore, the study also observed that the loss of p16Ink4a/p19Arf and Trp53 in adult acinar cells only contributed to the mPanIN and mPDAC development in the presence of K-Ras oncogenes, which may confirm the key role of K-ras in the carcinogenesis in the pancreas. Finally, oncogene-induced senescence, a natural defense mechanism against tumor development (39), was observed to be repressed by the inflammatory response of pancreatitis, and may be one of the mechanisms by which pancreatitis-induced inflammation contributes to PC, cooperating with simultaneous expression of the K-ras oncogene.

Recent studies described a new signaling pathway, the epidermal growth factor receptor (EGFR) signaling pathway, which was indicated to be required for K-ras-driven tumorigenesis and inflammation-associated tumorigenesis in the pancreas (40,41). In the mutant K-ras-driven PDA model, EGFR controls the differentiation of neoplastic precursors and induces tumor initiation, following which, EGFR promotes cancer progression by activating ERK. In addition, certain other signaling pathways are indicated in the participation of K-ras-induced pancreatic tumorigenesis, including the nuclear factor-κB (NF-κB) and Notch signaling pathways (42,43).

The K-ras oncogene is widely believed to be one of the initial components in pancreatic carcinogenesis, as of which several trials have been reported regarding the clinical applications (44,45). However, how the activated K-ras oncogenes are induced by the inflammatory microenvironment remains unclear. More notably, to elucidate the precise timing at which the K-ras mutations become detectable during the chronic inflammatory process will contribute to the early diagnosis of PC. Consequently, much more extensive work on the role of K-ras in pancreatic diseases will be involved in future studies.

### 3.2. COX-2

COX, also known as prostaglandin-endoperoxide synthetase, is the rate-limiting enzyme responsible for converting arachidonic acid to prostaglandins, leukotrienes and thromboxanes, which are considered to be the proinflammatory cytokines (46). Two isoforms of COX exist. COX-1 is constitutively expressed in numerous tissues and always plays the ‘house-keeping’ role in tissue homeostasis, whereas COX-2 is an inducible isoform observed to be overexpressed in numerous pathological changes, including inflammation and tumorigenesis (47).

COX-2 expression is elevated in response to a variety of proinflammatory stimuli, including interleukin-1 (IL-1), IL-6, tumor necrosis factor-α (TNF-α) and transforming growth factor-β (TGF-β) (48,49). Several studies have observed the overexpression of COX-2 in CP (25,50), and the COX-2 inhibitors attenuating the severity of acute pancreatitis in a rat model have indicated the correlation between COX-2 and the genesis of pancreatitis (51). Additionally, COX-2 has been found to be upregulated in PanIN (52), intraductal papillary mucinous neoplasm (IPMN) (53) and human PC tissue (53,54). Early suppression of COX-2 may contribute to preventing the progression of inflammatory and pre-malignant lesions to malignance in the pancreas (47–49). COX-2 promotes cell proliferation (58), inhibits apoptosis (59) and facilitates angiogenesis by increasing vascular endothelial growth factor (VEGF) production (60). Previously, a mouse model (61) was generated, in which the overexpression of COX-2 was under the control of a bovine keratin 5 promoter driving pancreatic acinar-to-ductal metaplasia, which revealed that the elevation of COX-2 was significantly correlated with the metaplasia-dysplasia progressing in the exocrine pancreas. However, the precise mechanism by which COX-2 promotes PC growth is unclear.

Thus, COX-2 conducts the definite linkage between inflammation and cancer in the pancreas. However, a number of laboratory and clinical studies, whose conclusions are controversial, have approached the therapeutic significance of targeting COX-2 activity for PC (62–67), and it requires more experimental evidence to confirm whether targeting COX-2 can be applied in clinical treatment.

### 3.3. NF-κB

NF-κB is a family of transcription factors, including NF-κB1, NF-κB2, Rel A, c-Rel and Rel B. These proteins are constitutively expressed in the cytoplasm of eukaryotic cells in an unactivated state as a result of combining with an inhibitory protein, inhibitor κB (IκB). The exact mechanism of NF-κB activation is complex and not completely clear. The impact of various pathogenic stimuli, including the proinflammatory factors TNF-α, IL-1 and bacterial lipopolysaccharide, enabling phosphorylation of IκBα subsequently results in the degradation of IκBα and exposures the sequence for DNA binding and nuclear translocation in NF-κB. Activated and nuclear translocated NF-κB, as a transcription factor, plays a significant role in the transcriptional control of certain inflammatory and cancer-related genes, including COX-2 (68), IL-8 (69), inducible NO synthase, cyclin D1 (68), c-Myc and VEGF. These molecules, by which NF-κB promotes PC growth, are upregulated and responsible for cell cycle control, angiogenesis and the inhibition of apoptosis in PC progression. Genetic mutations in NF-κB, IκB kinase (IKK) or upstream components of its signaling system have rarely been observed. Otherwise, exposure to the proinflammatory microenvironment resulting in activation of NF-κB in cancer has been widely hypothesized and demonstrated (70,71). The molecules for the activation of the IKK/NF-κB signaling pathway include growth factors, cytokines, lymphokines and microRNA (72), most of which may bridge the gap between inflammation and cancer.

Constitutive activation of NF-κB has been observed in several PC cell lines (73) and human PC tissue (74–76). The proinflammatory cytokine-paracrine loops established by inflammatory, immune and cancer cells potently activate NF-κB in PC (11,77). The autocrine secretion of IL-1α, induced by activator protein-1 activity, has been demonstrated to play a key role in the activation of NF-κB in metastatic PC cell lines (78). In turn, the activation of NF-κB can enhance expression of IL-1α, which results in a positive feedback loop for the constitutive NF-κB activation in PC. Additionally, IL-1α can enhance the expression of several NF-κB-regulated genes, including IL-8 and VEGF, which are responsible for the metastatic processes and angiogenesis of cancer, in PC cells (79). In conclusion, the IL-1α-NF-κB positive feedback loop makes great contributions to support the malignant phenotype in PC.

By contrast, previous studies have shown the essential role of the NF-κB pathway in K-ras-mutant PCs (43,80). As there have been less therapeutic strategies of PC-targeting on the K-ras signaling pathway, the inhibition of NF-κB is an attractive strategy for the treatment of K-ras-dependent PC.

### 3.4. SPINK1

SPINK1 has been identified as a trypsin inhibitor and is also known as a pancreatic secretory trypsin inhibitor (81). SPINK1 is secreted by the acinar cells of the exocrine pancreas into the pancreatic juice and is able to bind to trypsin to inhibit its activity. SPINK1 protects the pancreas from the impact of prematurely activated trypsinogen, and SPINK1 mutations, particularly the N34S mutation (82), have also been reported to lower the threshold for pancreatitis from other genetic or environmental factors (83) instead of initiating the development of CP.

SPINK1 mutations in patients with pancreatitis are firmly demonstrated in numerous studies (82,84–86). Notably, the intronic mutations, including N34S, IVS1-37T>C and IVS3-69insTTTT, do not affect the mRNA expression of the SPINK1 gene, and these mutations have not been observed to be capable of affecting the binding affinity and inhibitory activity of SPINK1 to trypsin. Ohmuraya *et al* (87) created a mouse model with deficient SPINK3 (a mouse homologue gene of human SPINK1) and showed that excessive autophagy in pancreatic acinar cells could be induced by the loss of the regulation of SPINK3, indicating that SPINK3 may act as a suppressor of autophagy. Therefore, the pathogenesis of pancreatitis may be more complex, and further studies will be required to elucidate the role of SPINK1 in the onset of pancreatitis.

Previously, Rebours *et al* (88) described the case of a CP-diagnosed patient with an N34S mutation of SPINK1. Cystic fibrosis transmembrane conductance regulator minor mutations were found in the multifocal moderate-dysplastic lesions of PanIN-2 in the duct epithelium following a left pancreatectomy. Shimosegawa *et al* (89) examined whether the SPINK1 gene N34S mutation could be a risk factor for PC in patients with CP. The study investigated the development of PC in three out of 16 CP patients with the N34S mutation (18.8%), while only three of 216 CP patients without the SPINK1 mutation (1.4%) developed PC, indicating that the N34S mutation of the SPINK1 gene may be a significant risk factor for the development of PC in patients with CP. By contrast, SPINK1 itself has been shown to have growth factor activity in various cell lines, including certain cancer cells (90,91). SPINK1 is described as a ligand for the EGFR. Ozaki *et al* (91) observed the increase in the cell numbers of PC cell lines following treatment with SPINK1. The results of their subsequent study demonstrate that SPINK1 stimulates the proliferation of PC cells through EGFR and its downstream signal molecules. The immunohistochemical study showed that SPINK1 and EGFR are co-expressed not only in pancreatic tubular adenocarcinoma, but also in PanINs (92). As EGFR is confirmed to be overexpressed in the tissues of CP and PC (93,94), and SPINK1 is produced by the acinar cells and is then secreted into the pancreatic duct, the interaction between SPINK1 and EGFR may be involved in the malignant transformation of normal pancreatic cells during the inflammatory process. A high level of SPINK1 expression has been reported to be associated with cancer progression, tumor recurrence and patient survival rates in certain other cancers, indicating that SPINK1 has additional functions in extrapancreatic cancers (95,96).

In summary, SPINK1 is described as an inhibitor of the onset of pancreatitis, and the mutation of SPINK1 is able to lower the threshold for pancreatitis from other genetic or environmental factors. In addition, SPINK1 itself can promote PC development through the EGFR pathway, while the other roles of SPINK1 in inflammatory incidence and tumorigenesis remain unknown. More laboratorial and clinical studies are required to support the role of SPINK1 between pancreatitis and PC.

### 3.5. ROS

ROS are highly reactive oxygen metabolites such as the superoxide radical (O_2_^−^) the hydroxyl radical (OH^−^) hydrogen peroxide (H_2_O_2_) and trioxygen (O_3_). ROS are produced in mitochondria as by-products of oxidative phosphorylation, and are part of the normal cellular metabolism. In inflammatory processes ROS are produced by phagocytes. Additionally, inflammatory cells may use cytokines, including TNF-α and TGF-β (97), to stimulate ROS accumulation in surrounding epithelial cells. In cancer cells, comparatively high levels of ROS can also result from increased metabolic activity, mitochondrial dysfunction, oncogene activity and the increased activity of oxidases, COXs and lipoxygenases (99,99).

ROS can oxidize lipids in the cell membrane, oxidatively modify protein, depolarize the mitochondrial membrane and induce DNA fragmentation to directly damage DNA and induce genomic instability (100). In addition to ROS-induced direct DNA damage, certain by-products produced from ROS-generated lipid peroxidation, including malondialdehyde (MDA) and 8-oxodeoxyguanosine, also known as oxidized DNA adducts, can be potent carcinogenic molecules (101). Varying levels of ROS always result in different effects on cell proliferation. ROS at acute high levels may act as potent cytotoxic molecules inducing cell apoptosis (102). Chronic low levels of ROS can affect genome stability and cause tumorigenesis (103,104), emphasizing the role of ROS in the initiation of malignancy in CP tissues.

Higher levels of oxidative DNA adducts and MDA have been found in patients with CP (105) and also in human pancreatic tumor tissue (106). Identically, the primary intracellular antioxidant enzymes, superoxide dismutase (SOD), catalase and glutathione peroxidase, are detected in the pancreas, and a gradually decreased expression of the three antioxidant enzymes has been shown in pancreatic cells from the normal pancreas to CP to PC (107). The upregulation of SODs can effectively suppress PC growth *in vitro* and *in vivo* (108). It has been indicated that chronic low levels of ROS promote malignant initiation and cancer cell proliferation in the pancreas. By contrast, ROS can active several signaling pathways, including the mitogen-activated protein kinase, PI3K/Akt NF-κB signaling, protein kinase C and p53 signaling pathways (109–111), which may tend to activate cell survival or cell death. ROS can participate in the apoptotic or anti-apoptotic mechanisms in oxidative stress-targeting therapeutics or oncogene-induced malignant transformation (112–115). Therefore, the actual role of ROS in pancreatitis-derived PC was really determined by the extent of accumulation and the regulation of activity. The balanced regulation of ROS in pancreatitis and PC should be further investigated, which may also be regarded as the potential therapy target for PC.

### 3.6. Proinflammatory cytokines

Proinflammatory cytokines that are secreted by immune and inflammatory cells are significant components in the inflammation and tumor microenvironment. Several cytokines, including IL-1, TNF, IL-6 and IL-23, are critical for inflammation and tumor growth. Varying cytokines affect certain downstream effectors, including NF-κB, activator protein-1, signal transducers and activators of transcription and SMAD transcription factors, to exhibit either promotion or inhibition effects in tumor progression (11,116). By contrast, oncoproteins, such as Ras and Myc, can also promote the secretion of proinflammatory cytokines, which have direct effects on cancer cell growth and survival (117). Altogether, proinflammatory cytokines significantly mediate the mechanisms of inflammation-related tumor initiation and promotion.

Significantly higher levels of TNF-α, IL-1, IL-6 and IL-8 have been observed in patients with CP and PC compared with healthy controls (118–121). IL-1 acting through the IL-1 receptor type I can promote the invasion and angiogenesis of PC cells (123). The cyst fluid IL-1 levels predict the risk of carcinoma in IPMN, indicating that IL-1 may facilitate malignant transformation in the pancreas (123). TNF-α is classically considered to be an inhibitor of the apoptosis of cancer cells by activating the expression of NF-κB (124–126). Additionally, the inflammatory-derived TNFα is able to stimulate migration and induce the epithelial-mesenchymal transition of human pancreatic carcinoma cells (127). IL-6 has been identified as a growth factor that enhances PC cell proliferation (118,128,129). An elevated IL-6 level is correlated with advanced PC, which is represented by cachexia and a poor-life status (130). IL-8, also known as chemokine (C-X-C motif) ligand 8 (CXCL-8), is a CXC chemokine that is produced by numerous types of cells in response to inflammatory stimuli. IL-8 plays a significant role in neutrophil chemotaxis and activation. In addition to its inflammatory role, IL-8 has been found to be an autocrine growth factor in PC (131,132). The expression of IL-8 is driven by NF-κB activation (133), and numerous cytokines, including TNF-α and leukemia inhibitory factor, can enhance the expression of IL-8 (131). IL-8 acts as a significant effector molecule bridging the gap between inflammation and cancer in the pancreas.

Recently, more cytokines have been identified that are correlated with the clinical stage and prognosis in patients with PC (134–136). Cytokines, as molecules derived from inflammatory cells and immune cells, should be highlighted as prospects for the future exploration of the molecular mechanism from inflammation to cancer in the pancreas.

## 4. Conclusion

Despite advances in oncology and surgery, patients with PC frequently have a poor performance and clinical prognosis. Clinical evidence has confirmed the correlation between inflammation and pancreatic tumorigenesis. Inflammatory responses play a significant role in PC initiation and progression. In turn, cancer-induced inflammatory microenvironment or secreted inflammatory molecules can enhance the proliferation of cancer cells and promote the inflammatory responses. Exploring the variation in molecular expression and function from inflammation to cancer may aid in the development of a route to reveal the causes and mechanisms of PC. Inflammation-targeted treatments and examinations exhibit an appealing perspective in the therapy and diagnosis (121) of inflammation-related cancer in the pancreas (135,137–139). In addition, great importance should be attached to the inflammatory responses in the process of assigning chemotherapies and surgeries for PC treatment. The treatment of inflammatory changes may be necessary for the preventive and therapeutic schedules of PC.

## References

[1] Siegel R, Ma J, Zou Z, Jemal A (2014). Cancer statistics, 2014. CA Cancer J Clin.

[2] Batty GD, Kivimaki M, Morrison D (2009). Risk factors for pancreatic cancer mortality: extended follow-up of the original Whitehall Study. Cancer Epidemiol Biomarkers Prev.

[3] Landi S (2009). Genetic predisposition and environmental risk factors to pancreatic cancer: A review of the literature. Mutat Res.

[4] Lowenfels AB, Maisonneuve P (2006). Epidemiology and risk factors for pancreatic cancer. Best Pract Res Clin Gastroenterol.

[5] Genkinger JM, Spiegelman D, Anderson KE (2009). Alcohol intake and pancreatic cancer risk: a pooled analysis of fourteen cohort studies. Cancer Epidemiol Biomarkers Prev.

[6] Hassan MM, Bondy ML, Wolff RA (2007). Risk factors for pancreatic cancer: case-control study. Am J Gastroenterol.

[7] Peek RM, Crabtree JE (2006). Helicobacter infection and gastric neoplasia. J Pathol.

[8] Eaden JA, Abrams KR, Mayberry JF (2001). The risk of colorectal cancer in ulcerative colitis: a meta-analysis. Gut.

[9] Canavan C, Abrams KR, Mayberry J (2006). Meta-analysis: colorectal and small bowel cancer risk in patients with Crohn’s disease. Aliment Pharmacol Ther.

[10] Solaymani-Dodaran M, Logan RF, West J, Card T, Coupland C (2004). Risk of oesophageal cancer in Barrett’s oesophagus and gastro-oesophageal reflux. Gut.

[11] Grivennikov SI, Greten FR, Karin M (2010). Immunity, inflammation, and cancer. Cell.

[12] Ekbom A, McLaughlin JK, Karlsson BM (1994). Pancreatitis and pancreatic cancer: a population-based study. J Natl Cancer Inst.

[13] Talamini G, Falconi M, Bassi C (1999). Incidence of cancer in the course of chronic pancreatitis. Am J Gastroenterol.

[14] Lowenfels AB, Maisonneuve P, Cavallini G, International Pancreatitis Study Group (1993). Pancreatitis and the risk of pancreatic cancer. N Engl J Med.

[15] Maisonneuve P, Lowenfels AB, Müllhaupt B (2005). Cigarette smoking accelerates progression of alcoholic chronic pancreatitis. Gut.

[16] Doll R, Peto R, Hall E, Wheatley K, Gray R (1994). Mortality in relation to consumption of alcohol: 13 years’ observations on male British doctors. BMJ.

[17] Bracci PM, Wang F, Hassan MM, Gupta S, Li D, Holly EA (2009). Pancreatitis and pancreatic cancer in two large pooled case-control studies. Cancer Causes Control.

[18] Bansal P, Sonnenberg A (1995). Pancreatitis is a risk factor for pancreatic cancer. Gastroenterology.

[19] Lowenfels AB, Maisonneuve P, DiMagno EP, Elitsur Y, Gates LK, Perrault J, Whitcomb DC, International Hereditary Pancreatitis Study Group (1997). Hereditary pancreatitis and the risk of pancreatic cancer. J Natl Cancer Inst.

[20] Chari ST, Mohan V, Pitchumoni CS, Viswanathan M, Madanagopalan N, Lowenfels AB (1994). Risk of pancreatic carcinoma in tropical calcifying pancreatitis: an epidemiologic study. Pancreas.

[21] Ji B, Tsou L, Wang H, Gaiser S (2009). Ras activity levels control the development of pancreatic diseases. Gastroenterology.

[22] Wang Z, Ali S, Banerjee S (2013). Activated K-Ras and INK4a/Arf deficiency promote aggressiveness of pancreatic cancer by induction of EMT consistent with cancer stem cell phenotype. J Cell Physiol.

[23] Bishop M, Xenoulis P, Steiner JM (2013). Association between this SPINK1 variant and clinically detectable pancreatitis. J Vet Intern Med.

[24] Hucl T, Jesnowski R, Pfützer RH, Elsässer HP, Löhr M (2007). SPINK1 variants in young-onset pancreatic cancer. J Gastroenterol.

[25] Schlosser W, Schlosser S, Ramadani M, Gansauge F, Gansauge S, Beger HG (2002). Cyclooxygenase-2 is overexpressed in chronic pancreatitis. Pancreas.

[26] Vinokurova LV, Berezina OI, Drozdov VN, Petrakov AV, Nilova TV (2011). Nitric oxide and indicators of oxidative stress in patients with exacerbation of chronic pancreatitis. Eksp Klin Gastroenterol.

[27] Bernards A, Settleman J (2004). GAP control: regulating the regulators of small GTPases. Trends Cell Biol.

[28] Voice JK, Klemke RL, Le A, Jackson JH (1999). Four human ras homologs differ in their abilities to activate Raf-1, induce transformation, and stimulate cell motility. J Biol Chem.

[29] Maekawa M, Nishida E, Tanoue T (2002). Identification of the anti-proliferative protein Tob as a MAPK substrate. J Biol Chem.

[30] Roux PP, Ballif BA, Anjum R, Gygi SP, Blenis J (2004). Tumor-promoting phorbol esters and activated Ras inactivate the tuberous sclerosis tumor suppressor complex via p90 ribosomal S6 kinase. Proc Natl Acad Sci USA.

[31] Rodriguez-Viciana P, Warne PH, Vanhaesebroeck B, Waterfield MD, Downward J (1996). Activation of phosphoinositide 3-kinase by interaction with Ras and by point mutation. EMBO J.

[32] Brognard J, Clark AS, Ni Y, Dennis PA (2001). Akt/protein kinase B is constitutively active in non-small cell lung cancer cells and promotes cellular survival and resistance to chemotherapy and radiation. Cancer Res.

[33] Rodriguez-Viciana P, Warne PH, Khwaja A (1997). Role of phosphoinositide 3-OH kinase in cell transformation and control of the actin cytoskeleton by Ras. Cell.

[34] Mulligan NJ, Yang S, Andry C, Klein M, O’Brien MJ (1999). The role of p21ras in pancreatic neoplasia and chronic pancreatitis. Hum Pathol.

[35] Mu DQ, Peng YS, Xu QJ (2004). Values of mutations of K-ras oncogene at codon 12 in detection of pancreatic cancer: 15-year experience. World J Gastroenterol.

[36] Guerra C, Schuhmacher AJ, Cañamero M (2007). Chronic pancreatitis is essential for induction of pancreatic ductal adenocarcinoma by K-Ras oncogenes in adult mice. Cancer Cell.

[37] Logsdon CD, Ji B (2009). Ras activity in acinar cells links chronic pancreatitis and pancreatic cancer. Clin Gastroenterol Hepatol.

[38] Guerra C, Collado M, Navas C (2011). Pancreatitis-induced inflammation contributes to pancreatic cancer by inhibiting oncogene-induced senescence. Cancer Cell.

[39] Collado M, Serrano M (2010). Senescence in tumours: evidence from mice and humans. Nat Rev Cancer.

[40] Navas C, Hernández-Porras I, Schuhmacher AJ, Sibilia M, Guerra C, Barbacid M (2012). EGF receptor signaling is essential for k-ras oncogene-driven pancreatic ductal adenocarcinoma. Cancer Cell.

[41] Ardito CM, Grüner BM, Takeuchi KK (2012). EGF receptor is required for KRAS-induced pancreatic tumorigenesis. Cancer Cell.

[42] Wang Z, Banerjee S, Ahmad A (2011). Activated K-ras and INK4a/Arf deficiency cooperate during the development of pancreatic cancer by activation of Notch and NF-κB signaling pathways. PLoS One.

[43] Daniluk J, Liu Y, Deng D (2012). An NF-κB pathway-mediated positive feedback loop amplifies Ras activity to pathological levels in mice. J Clin Invest.

[44] Friday BB, Adjei AA (2005). K-ras as a target for cancer therapy. Biochim Biophys Acta.

[45] Kamisawa T, Takuma K, Tabata T, Egawa N, Yamaguchi T (2011). Long-term follow-up of chronic pancreatitis patients with K-ras mutation in the pancreatic juice. Hepatogastroenterology.

[46] Prescott SM, Fitzpatrick FA (2000). Cyclooxygenase-2 and carcinogenesis. Biochim Biophys Acta.

[47] Williams CS, Mann M, DuBois RN (1999). The role of cyclooxygenases in inflammation, cancer, and development. Oncogene.

[48] Diaz A, Chepenik KP, Korn JH, Reginato AM, Jimenez SA (1998). Differential regulation of cyclooxygenases 1 and 2 by interleukin-1 beta, tumor necrosis factor-alpha, and transforming growth factor-beta 1 in human lung fibroblasts. Exp Cell Res.

[49] Aoki H, Ohnishi H, Hama K (2007). Cyclooxygenase-2 is required for activated pancreatic stellate cells to respond to proinflammatory cytokines. Am J Physiol Cell Physiol.

[50] Koliopanos A, Friess H, Kleeff J, Roggo A, Zimmermann A, Büchler MW (2001). Cyclooxygenase 2 expression in chronic pancreatitis: correlation with stage of the disease and diabetes mellitus. Digestion.

[51] O’Brien G, Shields CJ, Winter DC, Dillon JP, Kirwan WO, Redmond HP (2005). Cyclooxygenase-2 plays a central role in the genesis of pancreatitis and associated lung injury. Hepatobiliary Pancreat Dis Int.

[52] Albazaz R, Verbeke CS, Rahman SH, McMahon MJ (2005). Cyclooxygenase-2 expression associated with severity of PanIN lesions: a possible link between chronic pancreatitis and pancreatic cancer. Pancreatology.

[53] Niijima M, Yamaguchi T, Ishihara T, Hara T, Kato K, Kondo F, Saisho H (2002). Immunohistochemical analysis and in situ hybridization of cyclooxygenase-2 expression in intraductal papillary-mucinous tumors of the pancreas. Cancer.

[54] Molina MA, Sitja-Arnau M, Lemoine MG, Frazier ML, Sinicrope FA (1999). Increased cyclooxygenase-2 expression in human pancreatic carcinomas and cell lines: growth inhibition by nonsteroidal anti-inflammatory drugs. Cancer Res.

[55] Kokawa A, Kondo H, Gotoda T (2001). Increased expression of cyclooxygenase-2 in human pancreatic neoplasms and potential for chemoprevention by cyclooxygenase inhibitors. Cancer.

[56] Hillion J, Smail SS, Di Cello F (2012). The HMGA1-COX-2 axis: a key molecular pathway and potential target in pancreatic adenocarcinoma. Pancreatology.

[57] Kirane A, Toombs JE, Ostapoff K (2012). Apricoxib, a novel inhibitor of COX-2, markedly improves standard therapy response in molecularly defined models of pancreatic cancer. Clin Cancer Res.

[58] Kong G, Kim EK, Kim WS (2002). Role of cyclooxygenase-2 and inducible nitric oxide synthase in pancreatic cancer. J Gastroenterol Hepatol.

[59] Ding XZ, Tong WG, Adrian TE (2000). Blockade of cyclooxygenase-2 inhibits proliferation and induces apoptosis in human pancreatic cancer cells. Anticancer Res.

[60] Eibl G, Bruemmer D, Okada Y (2003). PGE(2) is generated by specific COX-2 activity and increases VEGF production in COX-2-expressing human pancreatic cancer cells. Biochem Biophys Res Commun.

[61] Colby JK, Klein RD, McArthur MJ (2008). Progressive metaplastic and dysplastic changes in mouse pancreas induced by cyclooxygenase-2 overexpression. Neoplasia.

[62] Furukawa F, Nishikawa A, Lee IS (2003). A cyclooxygenase-2 inhibitor, nimesulide, inhibits postinitiation phase of N-nitrosobis(2-oxopropyl)amine-induced pancreatic carcinogenesis in hamsters. Int J Cancer.

[63] Anderson KE, Johnson TW, Lazovich D, Folsom AR (2002). Association between nonsteroidal anti-inflammatory drug use and the incidence of pancreatic cancer. J Natl Cancer Inst.

[64] Wenger FA, Kilian M, Achucarro P (2002). Effects of Celebrex and Zyflo on BOP-induced pancreatic cancer in Syrian hamsters. Pancreatology.

[65] Wei D, Wang L, He Y, Xiong HQ, Abbruzzese JL, Xie K (2004). Celecoxib inhibits vascular endothelial growth factor expression in and reduces angiogenesis and metastasis of human pancreatic cancer via suppression of Sp1 transcription factor activity. Cancer Res.

[66] Jacobs EJ, Connell CJ, Rodriguez C (2004). Aspirin use and pancreatic cancer mortality in a large United States cohort. J Natl Cancer Inst.

[67] Schernhammer ES, Kang JH, Chan AT (2004). A prospective study of aspirin use and the risk of pancreatic cancer in women. J Natl Cancer Inst.

[68] Yamamoto Y, Gaynor RB (2001). Therapeutic potential of inhibition of the NF-kappaB pathway in the treatment of inflammation and cancer. J Clin Invest.

[69] Shi Q, Le X, Abbruzzese JL (1999). Cooperation between transcription factor AP-1 and NF-kappaB in the induction of interleukin-8 in human pancreatic adenocarcinoma cells by hypoxia. J Interferon Cytokine Res.

[70] Karin M, Cao Y, Greten FR, Li ZW (2002). NF-kappaB in cancer: from innocent bystander to major culprit. Nat Rev Cancer.

[71] Karin M (2006). Nuclear factor-kappaB in cancer development and progression. Nature.

[72] Lu Z, Li Y, Takwi A (2011). miR-301a as an NF-κB activator in pancreatic cancer cells. EMBO J.

[73] Liptay S, Weber CK, Ludwig L (2003). Mitogenic and antiapoptotic role of constitutive NF-kappaB/Rel activity in pancreatic cancer. Int J Cancer.

[74] Fujioka S, Sclabas GM, Schmidt C (2003). Function of nuclear factor kappaB in pancreatic cancer metastasis. Clin Cancer Res.

[75] Hoffmann A, Baltimore D (2006). Circuitry of nuclear factor kappaB signaling. Immunol Rev.

[76] Qing G, Xiao G (2005). Essential role of IkappaB kinase alpha in the constitutive processing of NF-kappaB2 p100. J Biol Chem.

[77] Carbone C, Melisi D (2012). NF-κB as a target for pancreatic cancer therapy. Expert Opin Ther Targets.

[78] Niu J, Li Z, Peng B, Chiao PJ (2004). Identification of an autoregulatory feedback pathway involving interleukin-1alpha in induction of constitutive NF-kappaB activation in pancreatic cancer cells. J Biol Chem.

[79] Melisi D, Niu J, Chang Z (2009). Secreted interleukin-1alpha induces a metastatic phenotype in pancreatic cancer by sustaining a constitutive activation of nuclear factor-kappaB. Mol Cancer Res.

[80] Barbie DA, Tamayo P, Boehm JS (2009). Systematic RNA interference reveals that oncogenic KRAS-driven cancers require TBK1. Nature.

[81] Kazal LA, Spicer DS, Brahinsky RA (1948). Isolation of a crystalline trypsin inhibitor-anticoagulant protein from pancreas. J Am Chem Soc.

[82] Witt H, Luck W, Hennies HC (2000). Mutations in the gene encoding the serine protease inhibitor, Kazal type 1 are associated with chronic pancreatitis. Nat Genet.

[83] Schneider A, Barmada MM, Slivka A, Martin JA, Whitcomb DC (2004). Clinical characterization of patients with idiopathic chronic pancreatitis and SPINK1 mutations. Scand J Gastroenterol.

[84] Chen JM, Mercier B, Audrezet MP (2001). Mutations of the pancreatic secretory trypsin inhibitor (PSTI) gene in idiopathic chronic pancreatitis. Gastroenterology.

[85] Pfützer RH, Barmada MM, Brunskill AP (2000). SPINK1/PSTI polymorphisms act as disease modifiers in familial and idiopathic chronic pancreatitis. Gastroenterology.

[86] Kuwata K, Hirota M, Nishimori I, Otsuki M, Ogawa M (2003). Mutational analysis of the pancreatic secretory trypsin inhibitor gene in familial and juvenile pancreatitis in Japan. J Gastroenterol.

[87] Ohmuraya M, Hirota M, Araki M (2005). Autophagic cell death of pancreatic acinar cells in serine protease inhibitor Kazal type 3-deficient mice. Gastroenterology.

[88] Rebours V, Couvelard A, Sauvanet A (2010). Pancreatic intraepithelial neoplasia is associated with chronic pancreatitis due to serine protease inhibitor kazal type 1 and cystic fibrosis transmembrane conductance regulator mutations. Pancreas.

[89] Shimosegawa T, Kume K, Satoh K (2009). Chronic pancreatitis and pancreatic cancer: prediction and mechanism. Clin Gastroenterol Hepatol.

[90] Ogawa M, Matsuura N, Higashiyama K, Mori T (1987). Expression of pancreatic secretory trypsin inhibitor in various cancer cells. Res Commun Chem Pathol Pharmacol.

[91] Ozaki N, Ohmuraya M, Hirota M (2009). Serine protease inhibitor Kazal type 1 promotes proliferation of pancreatic cancer cells through the epidermal growth factor receptor. Mol Cancer Res.

[92] Ozaki N, Ohmuraya M, Ida S (2013). Serine protease inhibitor Kazal type 1 and epidermal growth factor receptor are expressed in pancreatic tubular adenocarcinoma, intraductal papillary mucinous neoplasm, and pancreatic intraepithelial neoplasia. J Hepatobiliary Pancreat Sci.

[93] Oikawa T, Hitomi J, Kono A (1995). Frequent expression of genes for receptor tyrosine kinases and their ligands in human pancreatic cancer cells. Int J Pancreatol.

[94] Korc M, Friess H, Yamanaka Y (1994). Chronic pancreatitis is associated with increased concentrations of epidermal growth factor receptor, transforming growth factor alpha, and phospholipase C gamma. Gut.

[95] Tonouchi A, Ohtsuka M, Ito H (2006). Relationship between pancreatic secretory trypsin inhibitor and early recurrence of intrahepatic cholangiocarcinoma following surgical resection. Am J Gastroenterol.

[96] Gouyer V, Fontaine D, Dumont P (2008). Autocrine induction of invasion and metastasis by tumor-associated trypsin inhibitor in human colon cancer cells. Oncogene.

[97] Lo YY, Cruz TF (1995). Involvement of reactive oxygen species in cytokine and growth factor induction of c-fos expression in chondrocytes. J Biol Chem.

[98] Liou GY, Storz P (2010). Reactive oxygen species in cancer. Free Radic Res.

[99] Szatrowski TP, Nathan CF (1991). Production of large amounts of hydrogen peroxide by human tumor cells. Cancer Res.

[100] Leung PS, Chan YC (2009). Role of oxidative stress in pancreatic inflammation. Antioxid Redox Signal.

[101] Garcea G, Dennison AR, Steward WP, Berry DP (2005). Role of inflammation in pancreatic carcinogenesis and the implications for future therapy. Pancreatology.

[102] Lau AT, He QY, Chiu JF (2004). A proteome analysis of the arsenite response in cultured lung cells: evidence for in vitro oxidative stress-induced apoptosis. Biochem J.

[103] Lau AT, Chiu JF (2006). Proteomic and biochemical analyses of in vitro carcinogen-induced lung cell transformation: synergism between arsenic and benzo[a]pyrene. Proteomics.

[104] Barchowsky A, Dudek EJ, Treadwell MD, Wetterhahn KE (1996). Arsenic induces oxidant stress and NF-kappa B activation in cultured aortic endothelial cells. Free Radic Biol Med.

[105] Schoenberg MH, Birk D, Beger HG (1995). Oxidative stress in acute and chronic pancreatitis. Am J Clin Nutr.

[106] Kadlubar FF, Anderson KE, Häussermann S (1998). Comparison of DNA adduct levels associated with oxidative stress in human pancreas. Mutat Res.

[107] Cullen JJ, Mitros FA, Oberley LW (2003). Expression of antioxidant enzymes in diseases of the human pancreas: another link between chronic pancreatitis and pancreatic cancer. Pancreas.

[108] Teoh ML, Sun W, Smith BJ, Oberley LW, Cullen JJ (2007). Modulation of reactive oxygen species in pancreatic cancer. Clin Cancer Res.

[109] Benhar M, Engelberg D, Levitzki A (2002). ROS, stress-activated kinases and stress signaling in cancer. EMBO Rep.

[110] Martindale JL, Holbrook NJ (2002). Cellular response to oxidative stress: signaling for suicide and survival. J Cell Physiol.

[111] Leonard SS, Harris GK, Shi X (2004). Metal-induced oxidative stress and signal transduction. Free Radic Biol Med.

[112] Shimojo Y, Akimoto M, Hisanaga T (2013). Attenuation of reactive oxygen species by antioxidants suppresses hypoxia-induced epithelial-mesenchymal transition and metastasis of pancreatic cancer cells. Clin Exp Metastasis.

[113] DeNicola GM, Karreth FA, Humpton TJ (2011). Oncogene-induced Nrf2 transcription promotes ROS detoxification and tumorigenesis. Nature.

[114] Hossain MZ, Kleve MG (2011). Nickel nanowires induced and reactive oxygen species mediated apoptosis in human pancreatic adenocarcinoma cells. Int J Nanomedicine.

[115] Lau ST, Lin ZX, Leung PS (2010). Role of reactive oxygen species in brucein D-mediated p38-mitogen-activated protein kinase and nuclear factor-kappaB signalling pathways in human pancreatic adenocarcinoma cells. Br J Cancer.

[116] Lin WW, Karin M (2007). A cytokine-mediated link between innate immunity, inflammation, and cancer. J Clin Inves.

[117] Mantovani A, Allavena P, Sica A, Balkwill F (2008). Cancer-related inflammation. Nature.

[118] Wigmore SJ, Fearon KC, Sangster K, Maingay JP, Garden OJ, Ross JA (2002). Cytokine regulation of constitutive production of interleukin-8 and -6 by human pancreatic cancer cell lines and serum cytokine concentrations in patients with pancreatic cancer. Int J Oncol.

[119] Talar-Wojnarowska R, Gasiorowska A, Smolarz B (2009). Clinical significance of interleukin-6 (IL-6) gene polymorphism and IL-6 serum level in pancreatic adenocarcinoma and chronic pancreatitis. Dig Dis Sci.

[120] Miron N, Miron MM, Milea VG, Cristea V (2010). Proinflammatory cytokines: an insight into pancreatic oncogenesis. Roum Arch Microbiol Immunol.

[121] Dima SO, Tanase C, Albulescu R (2012). An exploratory study of inflammatory cytokines as prognostic biomarkers in patients with ductal pancreatic adenocarcinoma. Pancreas.

[122] Matsuo Y, Takeyama H, Guha S (2012). Cytokine network: new targeted therapy for pancreatic cancer. Curr Pharm Des.

[123] Maker AV, Katabi N, Qin LX (2011). Cyst fluid interleukin-1beta (IL1beta) levels predict the risk of carcinoma in intraductal papillary mucinous neoplasms of the pancreas. Clin Cancer Res.

[124] Wang CY, Mayo MW, Baldwin AS (1996). TNF- and cancer therapy-induced apoptosis: potentiation by inhibition of NF-kappaB. Science.

[125] Beg AA, Baltimore D (1996). An essential role for NF-kappaB in preventing TNF-alpha-induced cell death. Science.

[126] McDade TP, Perugini RA, Vittimberga FJ, Carrigan RC, Callery MP (1999). Salicylates inhibit NF-kappaB activation and enhance TNF-alpha-induced apoptosis in human pancreatic cancer cells. J Surg Res.

[127] Baran B, Bechyne I, Siedlar M (2009). Blood monocytes stimulate migration of human pancreatic carcinoma cells in vitro: the role of tumour necrosis factor - alpha. Eur J Cell Biol.

[128] Feurino LW, Zhang Y, Bharadwaj U (2007). IL-6 stimulates Th2 type cytokine secretion and upregulates VEGF and NRP-1 expression in pancreatic cancer cells. Cancer Biol Ther.

[129] Block KM, Hanke NT, Maine EA, Baker AF (2012). IL-6 stimulates STAT3 and Pim-1 kinase in pancreatic cancer cell lines. Pancreas.

[130] Ebrahimi B, Tucker SL, Li D, Abbruzzese JL, Kurzrock R (2004). Cytokines in pancreatic carcinoma: correlation with phenotypic characteristics and prognosis. Cancer.

[131] Kamohara H, Takahashi M, Ishiko T, Ogawa M, Baba H (2007). Induction of interleukin-8 (CXCL-8) by tumor necrosis factor-alpha and leukemia inhibitory factor in pancreatic carcinoma cells: Impact of CXCL-8 as an autocrine growth factor. Int J Oncol.

[132] Hussain F, Wang J, Ahmed R (2010). The expression of IL-8 and IL-8 receptors in pancreatic adenocarcinomas and pancreatic neuroendocrine tumours. Cytokine.

[133] Le X, Shi Q, Wang B (2000). Molecular regulation of constitutive expression of interleukin-8 in human pancreatic adenocarcinoma. J Interferon Cytokine Res.

[134] Xu X, Wang Y, Chen J (2012). High expression of CX3CL1/CX3CR1 axis predicts a poor prognosis of pancreatic ductal adenocarcinoma. J Gastrointest Surg.

[135] Grote VA, Kaaks R, Nieters A (2012). Inflammation marker and risk of pancreatic cancer: a nested case-control study within the EPIC cohort. Br J Cancer.

[136] Schmieder A, Multhoff G, Radons J (2012). Interleukin-33 acts as a pro-inflammatory cytokine and modulates its receptor gene expression in highly metastatic human pancreatic carcinoma cells. Cytokine.

[137] Bonifazi M, Gallus S, Bosetti C (2010). Aspirin use and pancreatic cancer risk. Eur J Cancer Prev.

[138] Bradley MC, Hughes CM, Cantwell MM (2010). Non-steroidal anti-inflammatory drugs and pancreatic cancer risk: a nested case-control study. Br J Cancer.

[139] Rothwell PM, Fowkes FG, Belch JF (2011). Effect of daily aspirin on long-term risk of death due to cancer: analysis of individual patient data from randomised trials. Lancet.

